# Combined therapy and antivascular endothelial growth factor monotherapies for polypoidal choroidal vasculopathy: A protocol for the systematic review and network meta-analysis of efficacy and safety

**DOI:** 10.1097/MD.0000000000013775

**Published:** 2018-12-21

**Authors:** Yun Zhang, Xun Li, Xiaoye Chen, Zhaolun Cai, Zirong Zhang, You Tang, Tiancong Chang, Misha Chen, Meixia Zhang

**Affiliations:** a Department of Ophthalmology, West China Hospital; b West China School of Medicine; c Department of Gastrointestinal Surgery, West China Hospital, Sichuan University, Chengdu, Sichuan; d Department of Ophthalmology, NO. 2 People's Hospital of Yunnan Province, Kunming, Yunnan, China; e Department of West China Medicine Technology Transfer Center, West China Hospital, Sichuan University, Chengdu, Sichuan, China.

**Keywords:** antivascular endothelial growth factor monotherapy, combined therapy, network meta-analysis, photodynamic therapy, polypoidal choroidal vasculopathy

## Abstract

**Background:**

Different antivascular endothelial growth factor (VEGF) monotherapy regimens and photodynamic therapy (PDT) combined with anti-VEGF therapy are available for patients with polypoidal choroidal vasculopathy (PCV). However, the comparative efficacy and safety of different anti-VEGF monotherapy regimens and combined therapy with PDT and anti-VEGF remains unknown. The aim of our study is to evaluate the efficacy and safety of anti-VEGF monotherapies and combined therapy in patients with PCV.

**Methods:**

We will systematically search PubMed, Embase, and the Cochrane library for eligible studies. The Cochrane Collaboration's tool for assessing the risk of bias in a randomized trial and the ROBINS-I tool will be used to assess the risk of bias in the included studies. The primary outcome is the mean change in best corrected visual acuity from baseline. The secondary outcomes are the mean change in central retinal thickness from baseline and the number of serious adverse events.

**Results:**

The result will generate a comprehensive suggestion for the treatment of PCV.

**Conclusion:**

The results of the network meta-analysis will be submitted in a peer-reviewed journal for publication.

**Ethics and dissemination:**

The study does not involve human subjects and requires no ethical approval or patient consent. The results of the network meta-analysis will be submitted in a peer-reviewed journal for publication and generate a comprehensive suggestion for the treatment of PCV.

## 1 Introduction

Polypoidal choroidal vasculopathy (PCV), which was first described and reported by Yannuzzi et al in the 1980s,^[1]^ has been generally acknowledged in recent years as a clinical subtype of age-related macular degeneration (AMD).^[2]^ In general, PCV is more prevalent in particular racial groups, especially in Asian individuals, and the prevalence of PCV accounts for 22.3% to 61.6% of patients diagnosed with exudative AMD.^[3–5]^ The pooled prevalence of PCV in white individuals with exudative AMD is 8.7%.^[6]^ Although the pathogenesis and genetic risk factors for PCV remain unclear, a missense variant in FGD6 confers an increased risk of PCV.^[7]^ PCV is clinically characterized by an abnormal vascular network of subretinal pigment epithelium of choroidal origin with aneurysmal dilatations, retinal pigment epithelial detachment, lipid exudation and recurrent, large subretinal hemorrhages^[8,9]^ and can cause serious and even permanent vision loss in people younger than those with classic exudative AMD.^[6]^ The gold standard for diagnosing PCV is polypoid dilations and abnormal branching of the vascular network presented in indocyanine green angiography.^[10]^ Currently, the main therapies for PCV include photodynamic therapy (PDT), anti-VEGF agents, and combined therapy using anti-VEGF and PDT.

PDT has been demonstrated to be an effective and selective strategy to regress polypoidal retinal lesions and to improve visual acuity.^[11]^ In addition, fewer PDT treatments per year were needed for PCV patients to obtain visual acuity than were for cases of wet AMD.^[12]^ The polyps and exudative lesions can regress with PDT therapy. However, the incomplete occluded branching vascular network could be a residual lesion, resulting in leakage and inducing choroidal neovascularization. The complications after PDT, such as hemorrhage-affected vision and choroidal ischemia, increased VEGF expression.^[13]^

The high expression of VEGF agents in the aqueous humor and vascular endothelium of PCV patients establishes the biological foundation of anti-VEGF therapy,^[14]^ which includes ranibizumab (Lucentis; Genentech, Inc., South San Francisco, CA), bevacizumab (Avastin; Genentech, Inc., South San Francisco, CA), and recently, aflibercept (Eylea, Regeneron, Tarrytown, NY, and Bayer HealthCare, Berlin, Germany). Anti-VEGF therapy performs well for improving visual function, reducing retinal thickness, and reducing levels of VEGF. However, the deficiency of anti-VEGF monotherapy is the regression of polyps, which was confirmed by the EVEREST study.^[15]^ The reason for different responsivity could be different balances of VEGF agents and angiogenic epithelium-derived growth factor levels.^[16]^ Therefore, to maximize visual acuity outcome and polypoidal lesion regression, combined therapy with PDT and anti-VEGF is widely used in clinical practice to produce the synergistic effects of angio-occlusion and antiangiogenesis.^[17]^

However, the most effective treatment for PCV has not yet been confirmed because of the shortage of head-to-head randomized controlled trials and the limitations of traditional meta-analyses. Hence, we plan to perform a systematic review and network meta-analysis to evaluate the efficacy and safety of each therapy alone and PDT in combination with anti-VEGF.

## 2 Method

This study will conform to the PRISMA-NMA^[18]^ checklist and will be performed based on the established protocol (PROSPERO: CRD42018104619). Ethical approval is not necessary because this study is based on aggregate data and does not involve humans.

### 2.1 Eligibility criteria

The PICOS strategy (patients, intervention, comparisons, outcome, study design type) determines the eligibility criteria for this study.

#### 2.1.1 Patients and comparison of interventions

Studies that contain patients with PCV treated by different anti-VEGF monotherapies and combined therapy with PDT and anti-VEGF will be included. Studies that provide insufficient data on the mean change in best corrected visual acuity (BCVA) from baseline will be excluded. There are no limitations on age, gender, and ethnic distribution.

#### 2.1.2 Outcomes

The primary outcome is the mean change in BCVA from baseline. The secondary outcomes are the mean change in central retinal thickness from baseline and the number of serious adverse events.

#### 2.1.3 Study design

The present study will evaluate published randomized and nonrandomized controlled trials comparing different anti-VEGF monotherapies and combined therapy using PDT and anti-VEGF for the treatment of PCV patients.

### 2.2 Information sources and search strategy

The PubMed, Embase, and the Cochrane library will be systematically searched for eligible trials through July 15, 2018. We will identify additional studies in the reference catalog of relevant studies.

Search strategy of PubMed was as follows:

**#1** (((((ranibizumab) OR bevacizumab) OR aflibercept)) OR photodynamic therapy) OR verteporfin for polypoidal
**#2** (PCV) OR polypoidal choroidal vasculopathy
**#3** #1 AND #2

### 2.3 Selection process and data management

Two reviewers will independently select and screen the title and abstract of each retrieved study according to the eligibility criteria and extract the relevant information and data from the included studies using Microsoft Excel 2016. The selection process summary will be based on the PRISMA flow diagram. The data will include the study features, patients’ characteristics, data needed for quality assessment, and outcome indicators. The patient's characteristics will contain the types of inventions received, drug dosages, therapeutic regimens, mean age, sample size, and outcome indicators.

### 2.4 Risk of bias of individual studies

Two investigators will independently evaluate the risk of bias of each selected RCT and nonrandomized studies using the Cochrane Collaboration's tool and ROBINS-I tool, respectively.^[19,20]^ Disagreements will be discussed and resolved with a third reviewer.

### 2.5 Data synthesis and statistical analysis

#### 2.5.1 Measures of treatment effects

Different measures will be used to evaluate the same outcomes. Continuous outcomes will be analyzed by calculating the weighted mean difference (WMD), and dichotomous outcomes will be pooled with an odds ratio (OR). The result of the network meta-analysis will be presented with WMD or OR and relative 95% CIs for each possible treatment. Heterogeneity will be assessed with the *Q*-statistic and *I*^2^ index. If *P* < .1 or *I*^2^ > 50%, it presents moderate heterogeneity at least, and the random-effects model should be used.^[21,22]^

### 2.6 Data analysis

First, traditional pairwise meta-analyses of all outcomes and comparisons at each time point will be performed using the random-effects model. Each head-to-head comparison will involve 2 RCTs at least.^[23]^ In the absence of head-to-head evidence, an indirect treatment comparison meta-analysis will be used to retrieve indirect, available evidence. Then, a network meta-analysis with a Bayesian random-effects model will be performed in a frequentist framework assuming equal heterogeneity parameters in all comparisons and considering the correlation caused by the multiarm studies.^[24]^ Except for pooled WMDs or ORs with 95% CIs, all relative ranking probabilities of different anti-VEGF monotherapies, PDT monotherapy, and combined therapy will be presented as surface under the cumulative ranking curve (SUCRA) values. The probability of the most effective treatment (first, second, third, and so on) will be calculated. The SUCRA analysis is used to summarize and report the probability values, which is a simple average rank transformation to provide a hierarchical treatment to reflect the location and variance of therapeutic effects. The larger the SUCRA value, the better the rank of intervention, with a maximum SUCRA of 1.0.^[25,26]^

The consistency between direct and indirect evidence in the network will be assessed by the node splitting method.^[27]^ There is no significant difference between direct and indirect evidence when 95% CIs of inconsistency factors are zero or *P* > .05.^[27]^

A funnel plot of sample and effect size will be drawn to determine whether there is a publication bias in the network meta-analysis. The contour-enhanced funnel plot will be used to help interpret the funnel scatter plot.^[28,29]^ We also intend to generate the network geometry, a graphical presentation of evidence network, which is an essential item in network meta-analysis.^[18]^

We will employ Stata version 14. (Stata Corp, College Station, TX) and R v3.5.0 (gemtc package and rjags package) to perform the statistical analysis.

## 3 Discussion

Currently, the optimal therapy for PCV is still uncertain. Both anti-VEGF therapy, PDT, and combined therapy are all available for patients with PCV. To our knowledge, this study will be the first network meta-analysis in the field to comprehensively compare different therapies for PCV. We hope our work can obtain a ranking of multiple therapies and provide recommendations for ophthalmologists.

## Author contributions

**Conceptualization**: Meixia Zhang, Yun Zhang, Xun Li

**Investigation**: Xiaoye Chen, Zirong Zhang

**Methodology**: Meixia Zhang, Zhaolun Cai

**Writing – original draft**: Yun Zhang, You Tang, Tiancong Chang, Misha Chen

**Writing – review & editing**: Meixia Zhang, Xun Li, Zhaolun Cai

## References

[1] Yannuzzi LA Sorenson J Spaide RF Idiopathic polypoidal choroidal vasculopathy (IPCV). Retina (Philadelphia, Pa) 1990;32:1–8. 1693009

[2] Laude A Cackett PD Vithana EN Polypoidal choroidal vasculopathy and neovascular age-related macular degeneration: same or different disease? Prog Retin Eye Res 2010;29:19–29. 19854291 10.1016/j.preteyeres.2009.10.001

[3] Kokame GT Polypoidal choroidal vasculopathy—an important diagnosis to make with therapeutic implications. Retina (Philadelphia, Pa) 2012;32:1446–8. 10.1097/IAE.0b013e3182695bf8 22922845

[4] Honda S Matsumiya W Negi A Polypoidal choroidal vasculopathy: clinical features and genetic predisposition. Ophthalmologica 2014;231:59–74. 24280967 10.1159/000355488

[5] Iida T Polypoidal choroidal vasculopathy with an appearance similar to classic choroidal neovascularisation on fluorescein angiography. Br J Ophthalmol 2007;91:1103–4. 17709578 10.1136/bjo.2007.116160 PMC1954907

[6] Lorentzen TD Subhi Y Sørensen TL Prevalence of polypoidal choroidal vasculopathy in white patients with exudative age-related macular degeneration: systematic review and meta-analysis. Retina 2018;38:2363–71. 29059101 10.1097/IAE.0000000000001872

[7] Huang L Zhang H Cheng CY A missense variant in FGD6 confers increased risk of polypoidal choroidal vasculopathy. Nat Genet 2016;48:640–7. 27089177 10.1038/ng.3546

[8] Wong RL Lai TY Polypoidal choroidal vasculopathy: an update on therapeutic approaches. J Ophthalmic Vis Res 2013;8:359–71. 24653824 PMC3957043

[9] Wong CW Yanagi Y Lee WK Age-related macular degeneration and polypoidal choroidal vasculopathy in Asians. Prog Retin Eye Res 2016;53:107–39. 27094371 10.1016/j.preteyeres.2016.04.002

[10] Spaide RF Yannuzzi LA Slakter JS Indocyanine green videoangiography of idiopathic polypoidal choroidal vasculopathy. Retina (Philadelphia, Pa) 1994;15:100–10. 10.1097/00006982-199515020-00003 7542796

[11] Gomi F Ohji M Sayanagi K One-year outcomes of photodynamic therapy in age-related macular degeneration and polypoidal choroidal vasculopathy in Japanese patients. Ophthalmology 2008;115:141–6. 17582498 10.1016/j.ophtha.2007.02.031

[12] Yamashita A Shiraga F Shiragami C One-year results of reduced-fluence photodynamic therapy for polypoidal choroidal vasculopathy. Am J Ophthalmol 2010;149:465–71. 20042180 10.1016/j.ajo.2009.09.020

[13] Akaza E Yuzawa M Matsumoto Y Role of photodynamic therapy in polypoidal choroidal vasculopathy. Jpn J Ophthalmol 2007;51:270–7. 17660987 10.1007/s10384-007-0452-3

[14] Matsuoka M Ogata N Otsuji T Expression of pigment epithelium derived factor and vascular endothelial growth factor in choroidal neovascular membranes and polypoidal choroidal vasculopathy. Br J Ophthalmol 2004;88:809–15. 15148217 10.1136/bjo.2003.032466 PMC1772169

[15] Koh A Lee WK Chen LJ EVEREST study: efficacy and safety of verteporfin photodynamic therapy in combination with ranibizumab or alone versus ranibizumab monotherapy in patients with symptomatic macular polypoidal choroidal vasculopathy. Retina (Philadelphia, Pa) 2012;32:1453–64. 10.1097/IAE.0b013e31824f91e8 22426346

[16] Gao G Li Y Zhang D Unbalanced expression of VEGF and PEDF in ischemia-induced retinal neovascularization. FEBS Lett 2001;489:270–6. 11165263 10.1016/s0014-5793(01)02110-x

[17] Nowak-Sliwinska P van den Bergh H Sickenberg M Photodynamic therapy for polypoidal choroidal vasculopathy. Prog Retin Eye Res 2013;37:182–99. 24140257 10.1016/j.preteyeres.2013.09.003

[18] Hutton B Salanti G Caldwell DM The PRISMA extension statement for reporting of systematic reviews incorporating network meta-analyses of health care interventions: checklist and explanations. Ann Intern Med 2015;162:777–84. 26030634 10.7326/M14-2385

[19] Higgins JPT Altman DG Gøtzsche PC The Cochrane Collaboration's tool for assessing risk of bias in randomised trials. BMJ 2011;343:d5928. 22008217 10.1136/bmj.d5928 PMC3196245

[20] Sterne JAC Hernán MA Reeves BC ROBINS-I: a tool for assessing risk of bias in non-randomised studies of interventions. BMJ (Clinical research ed) 2016;355:i4919. 10.1136/bmj.i4919 PMC5062054 27733354

[21] Higgins JP Thompson SG Deeks JJ Measuring inconsistency in meta-analyses. BMJ 2003;327:557–60. 12958120 10.1136/bmj.327.7414.557 PMC192859

[22] Higgins JP Thompson SG Quantifying heterogeneity in a meta-analysis. Stat Med 2002;21:1539–58. 12111919 10.1002/sim.1186

[23] DerSimonian R Laird N Meta-analysis in clinical trials. Control Clin Trials 1986;7:177–88. 3802833 10.1016/0197-2456(86)90046-2

[24] Salanti G Indirect and mixed-treatment comparison, network, or multiple-treatments meta-analysis: many names, many benefits, many concerns for the next generation evidence synthesis tool. Res Synth Methods 2012;3:80–97. 26062083 10.1002/jrsm.1037

[25] Salanti G Ades AE Ioannidis JP Graphical methods and numerical summaries for presenting results from multiple-treatment meta-analysis: an overview and tutorial. J Clin Epidemiol 2011;64:163–71. 20688472 10.1016/j.jclinepi.2010.03.016

[26] van der Valk R Webers CA Lumley T A network meta-analysis combined direct and indirect comparisons between glaucoma drugs to rank effectiveness in lowering intraocular pressure. J Clin Epidemiol 2009;62:1279–83. 19716679 10.1016/j.jclinepi.2008.04.012

[27] Dias S Welton NJ Caldwell DM Checking consistency in mixed treatment comparison meta-analysis. Stat Med 2010;29:932–44. 20213715 10.1002/sim.3767

[28] Chaimani A Higgins JPT Mavridis D Graphical tools for network meta-analysis in STATA. PLoS ONE 2013;8:10.1371/journal.pone.0076654 PMC3789683 24098547

[29] Peters JL Sutton AJ Jones DR Contour-enhanced meta-analysis funnel plots help distinguish publication bias from other causes of asymmetry. J Clin Epidemiol 2008;61:991–6. 18538991 10.1016/j.jclinepi.2007.11.010

